# Crustose Coralline Algae and a Cnidarian Neuropeptide Trigger Larval Settlement in Two Coral Reef Sponges

**DOI:** 10.1371/journal.pone.0030386

**Published:** 2012-01-25

**Authors:** Steve Whalan, Nicole S. Webster, Andrew P. Negri

**Affiliations:** 1 School of Marine and Tropical Biology, James Cook University, Townsville, Queensland, Australia; 2 Australian Institute of Marine Science, Townsville, Queensland, Australia; Leibniz Center for Tropical Marine Ecology, Germany

## Abstract

In sessile marine invertebrates, larval settlement is fundamental to population maintenance and persistence. Cues contributing to the settlement choices and metamorphosis of larvae have important implications for the success of individuals and populations, but cues mediating larval settlement for many marine invertebrates are largely unknown. This study assessed larval settlement in two common Great Barrier Reef sponges, *Coscinoderma matthewsi* and *Rhopaloeides odorabile*, to cues that enhance settlement and metamorphosis in various species of scleractinian coral larvae. Methanol extracts of the crustose coralline algae (CCA), *Porolithon onkodes*, corresponding to a range of concentrations, were used to determine the settlement responses of sponge larvae. Cnidarian neuropeptides (GLW-amide neuropeptides) were also tested as a settlement cue. Settlement in both sponge species was approximately two-fold higher in response to live chips of CCA and optimum concentrations of CCA extract compared to 0.2 µm filtered sea water controls. Metamorphosis also increased when larvae were exposed to GLW-amide neuropeptides; *R. odorabile* mean metamorphosis reached 42.0±5.8% compared to 16.0±2.4% in seawater controls and in *C. matthewsi* mean metamorphosis reached 68.3±5.4% compared to 36.7±3.3% in seawater controls. These results demonstrate the contributing role chemosensory communication plays in the ability of sponge larvae to identify suitable habitat for successful recruitment. It also raises the possibility that larvae from distinct phyla may share signal transduction pathways involved in metamorphosis.

## Introduction

Larval settlement is intricately linked to population maintenance and persistence, so understanding the processes that influence settlement and recruitment is fundamental to the management and conservation of marine ecosystems. This is particularly true for sessile marine invertebrates where a mobile larval phase is largely responsible for distribution patterns, and with key larval settlement behaviours being a first step to recruitment success. Research on the settlement of sessile invertebrate larvae to coral reefs has understandably focused on corals, which often dominate these habitats [1]–[5]. Nevertheless, there is a limited knowledge of the specific cues that contribute to settlement of corals and this is even more uncertain for other sessile invertebrates such as sponges [6]. It is clear that the processes contributing to larval settlement are complex with apparent physical and chemical substrate specificities inducing settlement in some taxa [5] while for others settlement specificity appears less important [7].

Physical cues that contribute to settlement include complexity of surface micro-topography [8], [9] and orientation of settlement surface and incidence of light [10], [11]. Chemical cues are also implicated in larval settlement [12]. Microbial biofilms are common inducers for sessile invertebrates [13]–[15] with the age and composition of biofilms being influential in larval settlement [16]. Other chemical cues associated with conspecifics [17] and host symbionts [18] also contribute to larval settlement. Importantly, larval settlement is often linked to a hierarchy of cues associated with habitats that optimise settlement and therefore recruitment to populations [6], [11].

A number of crustose coralline algal (CCA) species can induce settlement in various species of coral [10], [19]–[21]. Moreover, extracts of CCA with ethanol (or methanol) have induced settlement in some coral species [19] with gene expression profiles during early metamorphosis being similar in coral larvae exposed to either live CCA or CCA extracts [22]. Although apparently common, this induction of coral larval metamorphosis by CCA is not universal, as the larvae of some coral species are not induced by the presence of CCA [21]. Nevertheless, the very clear settlement induction of key groups of corals to CCA, coupled with the ubiquitous presence of these algae on coral reefs, raises questions as to the importance of this habitat cue for other sessile coral reef invertebrates, such as sponges.

The identification of settlement cues associated with habitats suggests marine larvae have the ability to recognise specific compounds that either identify favourable habitats or initiate metamorphosis [15], [23]. For cnidarians with fundamental neural pathways, exposure to neuropeptides has been shown to initiate metamorphosis (and sometimes settlement) for different classes within this phylum [24]. A family of neuropeptides known as GLW-amides has been linked to signalling and internal coordination of metamorphosis in some cnidarians often following exposure to external environmental cues [25], [26]. Within the Scleractinia, synthetic analogues of the neuropeptide GLW-amide induces metamorphosis in the larvae of some Acroporid corals, but to date has not elicited responses in other coral genera tested [27].

Neuro-transmission signalling compounds and their role in larval metamorphosis appear conserved across classes of cnidarians [27], and it is also plausible that similar systems may operate in the closely related Porifera. Despite the notion that sponges exhibit no distinct neural capacity, recent work detailing the genome of the sponge, *Amphimedon queenslandica*, suggests sponges have the building blocks of neural genes [28]. Of interest, is that while some key genes related to synaptic function are missing in *A. queenslandica*, other genes found in metazoan sensory systems are embedded in the *A. queenslandica* genome [28]. Questions surrounding the complexity of these genes and how they might contribute to stimulating or initiating metamorphosis, as seen in the larvae of coral species, are as yet unexplored.

This study aimed to investigate whether cues commonly implicated in the settlement and metamorphosis of coral larvae also influences larval settlement for coral reef sponges. We specifically tested the settlement of two common species of Great Barrier Reef (GBR) sponges, *Cosocinoderma matthewsi* and *Rhopaloeides odorabile*, to the CCA *Porolithon onkodes*. This included both live algae and a range of concentrations of algal extracts. We also tested the potential morphogenic activity of a synthetic analogue of GLW-amide, a cnidarian neuropeptide, which can induce metamorphosis in several Acroporid coral species [27]. We found that larval settlement was enhanced in response to both CCA and GLW-amide neuropeptides adding to our knowledge of larval settlement for coral reef invertebrates.

## Methods

### Study sites and species

The common GBR sponges' *R. odorabile* and *C. matthewsi* were used in this study [29]. Substantial information central to their fundamental biology and ecology define them as excellent model species to explore the process of larval recruitment in coral reef sponges [6], [14], [30]–[32]. Both species have separate sexes, females brooding parenchymellae larvae with annual larval releases occurring over 4–5 weeks during the Austral summer [6], [14].

Ten female *C. matthewsi* were collected from the reef slope of Pioneer bay, Orpheus Island (18°35.61S, 146°29.05E) and transported to facilities at Orpheus Island Research Station (OIRS) where they were maintained in flow through aquaria during December 2010. Twelve female *R. odorabile* were collected from Rib Reef (18°29.51S, 146°52.70E) and transported to aquaria facilities at the Australian Institute of Marine Science (AIMS) in January 2011. Maintaining sponges in flow-through aquaria at OIRS and AIMS allowed controlled collection of larvae over several hours during their morning (*C. matthewsi*) and afternoon (*R. odorabile*) releases. Both species release larvae over several hours each day [6], [14].

Larvae were collected using larval traps, following Whalan et al. [6]. Briefly, mesh nets were placed over sponges, each trap housing a central collection jar that floated apically over the sponges. Larvae are positively phototactic at release with larvae congregating at the top of the jar, until collection. Larvae were collected over 2–4 hours, and pooled for use in experimental assays.

### Settlement assays

For all settlement assays, six well polystyrene cell culture plates were used (IWAKI). Treatments were randomised among wells of the plates and ten larvae were introduced into each treatment (n = 6 wells) by gentle pipetting. The final volume in each well was 10 ml, comprising the treatment concentration with the balance comprising 0.2 µm filtered sea water (FSW). Plates were maintained in shallow dishes with flow through sea water, acting as a water bath to maintain a consistent ambient temperature (≈28°C). Settlement was recorded at 2, 4, 6, 12, 24, 30 and 42 h for *R. odorabile* coinciding with the completion of larval settlement. The same time periods were also used for *C. matthewsi*, with the addition of 48 and 57 h to accommodate the completion of larval settlement.

The use of the term “settlement” can be ambiguous. Following Hadfield [15], settlement involves the transition from a planktonic to benthic life mode accompanied by processes facilitating attachment before metamorphosis; metamorphosis describes developmental changes where distinct and permanent morphological changes are undertaken to form a juvenile. Hereafter, terms of settlement and metamorphosis are based on Hadfield's [15] definition” and since permanent attachment was always observed during metamorphosis in these larvae we use the term “settlement” to encompass both processes.

### Effect of CCA

Samples of CCA (*Porolithon onkodes*) were collected from Bramble Reef (18°24.764S, 146°42.868E) and transported in flowing seawater to AIMS where they were maintained in flow through aquaria pending experimentation. This species was chosen because it is common to shallow reef habitats on the GBR and has been implicated in the settlement of larvae from several species of coral [21]. Small pieces (5×5 mm) of live CCA were used in assays. CCA was brushed lightly to remove any debris before being used in assays. To control for the carbonate substrate that CCA was attached to, 5×5 mm chips of sterile coral rubble were also tested.

To assess the effect of CCA, without the bias of attached coral rubble, *P.onkodes* was removed from the coral rubble substrate it grows on and methanol extracts of CCA were prepared. While our aim was to reduce the interference of ancillary cues associated with CCA (e.g. attached coral rubble) epiphytic material on the surface of CCA could be potentially included in the extraction process and this was an unavoidable artefact. Extracts were prepared by grinding 4 g wet mass of CCA in 10 volumes of methanol (HPLC grade). The slurry was allowed to sit for 24 h at 4°C then vacuum filtered through a GFF filter (Whatman). The methanol was evaporated to dryness under a stream of N_2_ then suspended with sonication in 2 ml MQ-water. This extract was applied to a 500 mg C18 SPE cartridge (Waters) and eluted with an additional 10 ml MQ-water under vacuum. This water wash was discarded and the active fraction eluted with 15 ml 4∶1 methanol: MQ-water (v/v). This active fraction was freeze dried and resuspended in 8 ml HPLC grade ethanol (a final extract concentration of 500 mg CCA ml^−1^) and stored frozen.

To test a concentration response of CCA extracts on larval settlement, extract volumes equivalent to 0.1, 0.3, 1 and 3 µl (extract) ml^−1^ (sea water), equivalent to CCA surface areas of 0.2, 0.6, 2 and 6 mm^2^ respectively, were used for assays. These volumes were added to empty wells and allowed to evaporate completely to dryness before seawater and larvae were added. Preliminary tests with higher extract concentrations (10 µl ml^−1^) resulted in high % mortality of larvae for both sponge species while no mortality was recorded for larvae at 3 µl ml^−1^. Controls included a treatment of 0.2 µm FSW and an ethanol control equivalent to the highest extraction concentration.

### Effect of GLW-amide neuropeptides

GLW-amide neuropeptides are linked to pathways that induce metamorphosis in larvae of several coral species [26], [27]. To test the effect of this cue on settlement of *C. matthewsi* and *R. odorabile*, a range of concentrations of GLW-amide neuropeptides were used. GLW-amide (sequence EPLPIGLWa) was purchased from Sigma Genosys and made up to a concentration of 1 mM in 0.2 µm filtered sea water (FSW). Six concentrations were tested in assays corresponding to 0.1, 0.3, 1, 3, 10 and 30 µM, in addition to a control of 0.2 µm FSW.

### Statistical treatment

Data are reported as means (±1 standard error). Statistical analyses were performed using SPSS v.17. Two approaches were followed, one analysis being the assessment of treatment effect (live CCA, CCA extracts and GLW-amide) on larval settlement over time. This required a repeated measures analysis of variance (RM ANOVA) that would allow interpretations of whether time to settlement increased or decreased in response to the different treatments.

A second approach assessed settlement at one final time point coinciding with the completion of larval settlement. This represents the settlement (metamorphosis) of larvae into a juvenile sponge and therefore the first stage towards recruitment to a population. Settlement had concluded by 42 h in *R. odorabile* and by 57 h in *C. matthewsi*. For this analysis, a one way analysis of variance (ANOVA) was undertaken on settlement among treatments. Tukey's HSD post hoc test was used to establish where significant differences occurred.

## Results

Larval settlement occurred between 6 and 57 h post-release in *C. matthewsi* and 12 and 42 h post-release in *R. odorabile*.

### CCA -live

**Figure 1:**
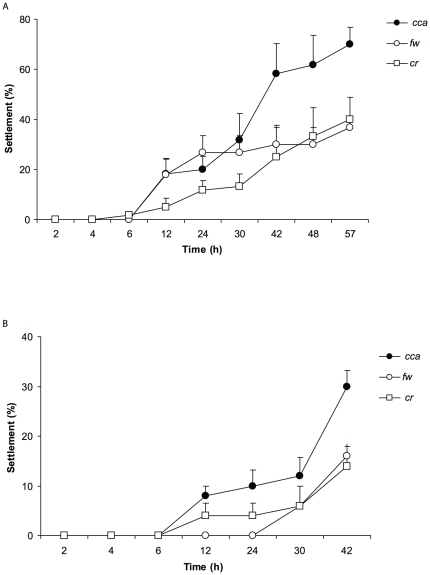
Larval settlement in response to live CCA. Mean percentage of (A) *C. matthewsi* and (B) *R. odorabile* larvae settled (+1SE) over time in response to live chips of the crustose coralline algae, *Porolithon onkodes* (CCA), in comparison to 0.2 µm filtered seawater controls (fw) and coral rubble (cr). n = 6 with 10 larvae per replicate.

*C. matthewsi*: In live CCA treatments larval settlement occurred on the surfaces of Petri dishes with no larvae settling on the CCA. A first analysis to examine whether larvae settled more rapidly in response to live CCA showed that both time and CCA had an effect on larval settlement and this was supported by a significant interaction of time and treatment (Table 1, Fig. 1A). The interactive effect of time and cue is clearly demonstrated from 42–57 h (Fig. 1A).Significant main effects of time and cue were also evident.

**Table 1:** Repeated measure ANOVA summary statistics of larval settlement in response to cues associated with the live crustose coralline algae, *Porolithon onkodes*(cue), over time.

Species	Variability	Source	df	MS	*F*	p
***C. matthewsi***	within subjects	Time	2.37	216.38	35.65	0.00
		time×cue	4.75	17.07	2.81	0.03
		Residual	35.52	6.07		
	between-subjects	cue	2.00	29.75	3.38	0.06
		Residual	15.00	8.79		
***R. odorabile***	within-subjects	Time	2.40	19.37	39.82	0.00
		time×cue	4.80	1.45	2.99	0.03
		Residual	28.78	0.49		
	between-subjects	cue	2.00	2.98	5.26	0.02
		Residual	12.00	0.57		

*F* values and significance are based on the Greenhouse- Geisser correction.

A second analysis ignoring cumulative settlement over time and focusing on total settlement at the completion of the experiment, showed a significant effect of live CCA on *C. matthewsi* larval settlement(ANOVA, *F_2,15_* = 7.34, p = 0.01). More specifically, significantly higher settlement occurred in response to CCA (70.0±6.8%) compared to FSW (36.7±3.3%) or coral rubble (40±8.9%), both of which showed consistent settlement (Fig. 1a, Tukey's HSD, p<0.05).

*R. odorabile*: As with larval assays of *C. matthewsi*, *R.odorabile* larvae did not settle on live CCA, but on the surfaces of Petri dishes. A significant interactive effect of time and cue were evident, in addition to significant main effects of time and cue on larval settlement (Table 1, Fig. 1B).

The second analysis for data associated with the single time point at 42 h showed a significant influence of live CCA on larval settlement (ANOVA, *F_2,12_* = 7.13, p = 0.01). Larval settlement was higher in live CCA treatments (30±2.9%) than FSW controls (16.0±2.4%) or treatments with coral rubble (14±4%) (Fig. 1B, Tukey's HSD, p<0.05). Larvae exhibited similar settlement success in both FSW and rubble treatments.

### CCA - extracts

**Figure 2:**
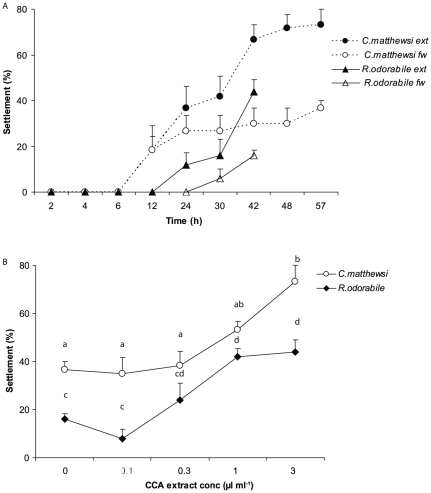
Larval settlement in response to CCA extracts. (A) Mean percentage of larvae settled (+1SE) in response to extracts from the CCA, *Porolithon onkodes*, equivalent to a concentration of 3 µl ml^−1^,and 0.2 µm filtered seawater controls (fw). (B) Mean percentage of larvae settled (+1SE) at four concentrations of CCA extract (µl extract ml^−1^ seawater) for *C. matthewsi* at 57 h post release and *R. odorabile* at 42 h post release. n = 6 with 10 larvae per replicate.

*C. matthewsi*: Analysis to determine whether larvae settled more rapidly in response to CCA extracts showed a significant interaction of time and cue, in addition to significant main effects of both factors (Table 2). For figure clarity, only data from one of the highest settlement responses (the 3 µl ml^−1^ treatment) is shown, which clearly demonstrates the trend of time×treatment, particularly over the period 12–57 h (Fig. 2A). Data analysed at the end time point of 57 h showed a significant effect of CCA extract concentration on larval settlement (Fig. 2 A–B, ANOVA, *F_5,30_* = 6.70, p = 0.00). Moreover, settlement at concentrations of 3 µl ml^−1^ was significantly higher than lower concentrations or FSW (Fig. 2B, Tukey's HSD, p<0.05).

**Table 2:** Repeated measure ANOVA summary statistics of larval settlement in response to cues associated with extracts of the crustose coralline algae, *Porolithon onkodes*(cue), over time.

Species	Variability	Source	df	MS	*F*	p
***C. matthewsi***	within subjects	Time	3.28	280.92	97.29	0.00
		time×cue	16.41	8.79	3.05	0.00
		residual	98.47	2.89		
	between-subjects	cue	5.00	27.28	3.45	0.01
		residual	30.00	7.91		
***R. odorabile***	within-subjects	time	2.35	68.99	73.27	0.00
		time×cue	11.76	4.45	4.73	0.00
		residual	56.43	0.94		
	between-subjects	cue	5.00	3.45	4.21	0.01
		residual	24.00	0.82		

*F* values and significance are based on the Greenhouse- Geisser correction.

*R. odorabile*: Analysis of time to settlement in response to CCA extract revealed that an interaction of both time and cue contributed to settlement (Table 2). Both main effects were also significant. Data from one of the highest settlement responses (3 µl ml^−1^) demonstrates the trend of time×treatment, particularly from 24–42 h (Fig. 2A). The second analysis, for data associated with the end point of the experiment (42 h),showed a significant effect of CCA extract concentration on larval settlement(Fig. 2 A–B, ANOVA, *F_5,24_* = 8.58, p = 0.00). Specifically, settlement for concentrations of 1 µl ml^−1^ and 3 µl ml^−1^ was 42±3.3% and 44±5.1% respectively, which was significantly higher than settlement associated with CCA extract concentrations of 0.1 µl ml^−1^ at 8.0±3.9% or FSW at 16.0±2.4% (Fig. 2B, Tukey's HSD, p<0.05).

### GLW-amide neuropeptides

#### *C. matthewsi*

**Figure 3:**
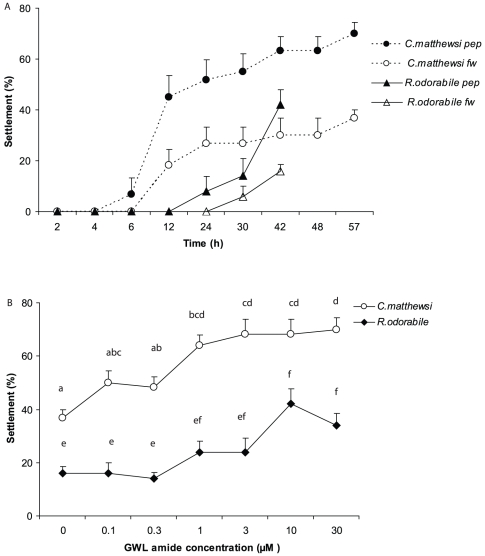
Larval settlement in response to a cnidarian neuropeptide. (A) Mean percentage of larvae settled (+1SE) in response to GLW-amide, equivalent to a concentration of 30 µM, and 0.2 µm filtered seawater controls (fw). (B) Mean percentage of larvae settled (+1SE) at six concentrations of GLW-amide for *C. matthewsi* at 57 h post release and *R. odorabile* at 42 h post release. n = 6 with 10 larvae per replicate.

Larval settlement profiles were similar to the CCA experiments where larval settlement occurred between 6–57 h (Fig. 3A). There was a significant interaction of time and cue on larval settlement in addition to significant main independent effects of time and cue (Table 3). Again for figure clarity, data from one of the highest settlement responses (30 µM) is presented, which demonstrates the trend of time×treatment, particularly over the period 12–57 h. When the final time point (57 h) was analysed separately, there was a significant influence of GLW-amide neuropeptides on settlement (ANOVA, *F_6,34_* = 8.29, p = 0.00). There were clear differences between settlement of mid to high concentrations of GLW-amide neuropeptides (3–30 µM) and settlement at 0.3 µM and FSW (Fig. 3B, Tukey's HSD, p<0.05). Moreover, there was consistent settlement at concentrations from 1 µM (64±4%) to 30 µM (70.0±4.5%), but settlement in these concentrations were significantly higher than FSW (36.7±3.3%). Settlement at concentrations from 0.1–0.3 µM were at similar levels to FSW.

**Table 3:** Repeated measure ANOVA summary statistics of larval settlement in response to cues associated with the cnidarian neuropeptide, GLW-amide (cue), over time.

Species	Variability	Source	df	MS	*F*	p
***C. matthewsi***	within subjects	time	3.00	584.43	162.45	0.00
		time×cue	18.00	6.75	1.87	0.03
		residual	102.00	3.60		
	between-subjects	cue	6.00	35.00	4.09	0.03
		residual	34.00	8.55		
***R. odorabile***	within-subjects	time	2.27	80.01	72.40	0.00
		time×cue	13.60	2.86	2.59	0.01
		residual	63.45	1.11		
	between-subjects	cue	6.00	1.49	1.88	0.12
		residual	28.00	0.80		

*F* values and significance are based on the Greenhouse- Geisser correction.

#### *R. odorabile*

Larval settlement in response to GLW-amide neuropeptides commenced between 12 and 24 h for all peptide treatments and from 30 h in the FSW treatment (Fig. 3A). A significant interaction of time to settlement and cue influenced larval settlement (Table 3). Data from one of the highest settlement responses (30 µM) demonstrates the trend of time×treatment, particularly from 24–42 h (Fig. 3A). The single time point analysis at the completion of the experiment (42 h) showed that GLW-amide neuropeptides had a significant effect on larval settlement (ANOVA, *F_6,28_* = 7.52, p = 0.00). More specifically, the highest settlement occurred in treatments at concentrations of 10 µM and 30 µM, which showed consistent mean settlement of 42±5.8% and 40±4.5% respectively, although mid concentrations of 1 and 3 µM were also similar at 24±4% and 24.0±5.8% respectively (Fig. 3B, Tukey's HSD, p<0.05). Notably, settlement at concentrations of 0.1–0.3 µM and FSW were below 20% and significantly less than higher concentrations of 10–30 µM.

## Discussion

Larval settlement in response to cues associated with CCA has not previously been documented in tropical sponges. CCA enhances larval settlement in many species of both hard and soft corals [33], [19] so the finding that there is a significant effect on larvae of *R. odorabile* and *C. matthewsi* is further recognition of the role CCA plays as a settlement cue for sessile coral reef invertebrates. CCA is common to most coral reef environments [34] with functional roles associated with reef accretion [35]. Furthermore, the ubiquitous presence of CCA on coral reefs coupled with its role in larval settlement processes further highlight the importance of these algae as a signal of reef habitat to recruiting larvae [36].

The ability of sessile invertebrate larvae to identify optimal environments to settle is critical given metamorphosis is irreversible. Therefore, settlement to adverse environments can have detrimental impacts on individuals and populations. Detailed information of the cues contributing to settlement in sponges is limited, but habitat related cues associated with biofilms are important for several species of sponges [6], [14]. Coral rubble also enhances the transition for initial settlement/attachment to metamorphosis in the sponge *R. odorabile* highlighting the importance of this substrate for post-settlement survival [6]. Settlement of both *C. matthewsi* and *R. odorabile* in response to live CCA is consistent with this proposal, with CCA acting as a signal that larvae have entered a coral reef habitat where post-settlement survival should be maximised.

Cues tested in the present study initiated higher levels of settlement in *C. matthewsi*, which exhibited an approximate twofold higher settlement than *R. odorabile*. The settlement success of sponge larvae upon release is similar to that reported for brooding corals [37]. Settlement success in newly released larvae may be related to their level of development upon release from the parent colony and this may differ between species. Another explanation for differential settlement between sponge species is that *R. odorabile* larvae are more specific in their settlement behaviours. *R. odorabile* larvae also show increased settlement to combinations of biofilm and coral rubble, in comparison to biofilms alone, suggesting a matrix of chemical cues may be required to optimise larval settlement in this species [6]. Importantly, both species showed increased settlement in comparison to FSW controls and coral rubble, supporting the premise that larvae can detect cues that mimic suitable habitats. The settlement to FSW controls without a cue may also provide support to the desperate larval hypothesis, whereby larvae become desperate to settle irrespective of cues [38]. Overall, these results reinforce the idea that there is a complicated matrix of settlement specificity among coral and sponge species [6], [7] and indeed between larvae from broadcast spawning versus brooding species which is exemplified in some coral species [7], [19].

Although recent data documenting sponge larval settlement in coral reefs has highlighted the importance of specific cues for habitat recognition and to optimise settlement (i.e. biofilms and coral rubble) it is focused on just a few studies [6], [11], [14]. The precise compounds that *C. matthewsi* and *R. odorabile* are responding to in CCA was not undertaken in this study, but coral larvae respond to a sulphated glycosaminoglycan [2] and to the bromotyrosine derivative 11-deoxyfistularin-3 [39]. Bacteria associated with CCA may also be a source of compounds like tetrabromopyrrole that induce metamorphosis in corals [40].

Although laboratory studies do not represent field conditions, they are highly suited to isolate the influence of a single factor on metamorphosis and to examine dose-response relationships. The enhanced settlement in response to CCA observed in laboratory studies here indicates sponge larvae possess chemosensory capabilities that play a role in the selection of microhabitats for recruitment. Chemosensory ability has been demonstrated for several groups of marine invertebrates, particularly for barnacles where sensory antennules are used to select optimal settlement sites [41]. Both *R. odorabile* and *C. matthewsi* exhibited enhanced settlement with increased concentrations of CCA extract as well as reduced settlement and increased mortality at the highest CCA extract concentration (10 µl extract ml^−1^ seawater). Reduced settlement and mortality have also been observed for coral larvae exposed to similarly elevated concentrations of CCA extracts and may reflect a toxic response caused by increased concentrations of co-extracted compounds in the semi-purified CCA extract [19].

The response of sponge larvae to the GLW-amide neuropeptides was a curious result, given the specificity of this cue for a different phylum. GLW-amide neuropeptides are associated with hydrozoans [25], and have induced metamorphosis in several coral species [26], [27]. Importantly, further work detailing more sponge species with reproducible results would be valuable before general comparisons can be appropriately made linking substrate cues that mediate settlement between these two phylum's or among classes of cnidarians. Of interest though is the specificity it elicits in metamorphosis, as demonstrated by inducing metamorphosis in some coral species but not others [27]. As for the CCA extract, GLW-amide neuropeptides elicited increased settlement responses at low to mid concentrations (1–10 µM), which is consistent with responses recorded for coral larvae [26]. Corals exposed to GLW-amide neuropeptides often undergo metamorphosis without attachment [26] and gene expression patterns in larvae exposed to GLW-amide neuropeptides indicates a role for this peptide class in signal transduction related to metamorphosis [22]. Until recently, our understanding of sponge neural systems was limited. The genome of *A. queenslandica*, however, has provided new insights into genes with neural links and sets the stage for recognising the potential for neural capacities in sponges [28]. More work is required to expand our understanding of how GLW-amide neuropeptides, and for that matter CCA, influence metamorphosis in *C. matthewsi* and *R. odorabile*, but these initial results provide a foundation to explore larval sensory abilities in the presence of natural bio-chemicals.
